# Zoledronic Acid May Reduce Intraoperative Bleeding in Spinal Tumors: A Prospective Cohort Study

**DOI:** 10.1155/2015/936307

**Published:** 2015-01-22

**Authors:** Juan Wu, Wei Zheng, Yan Tan, Xiao-Yuan Hu, Quan Huang, Kai-Hua Fan, Jie Ma, Wen-Jing Xiao, Jian-Dong Ren, Jun Hou, Jian-Ru Xiao

**Affiliations:** ^1^Department of Pharmacy, Chengdu Military General Hospital, Chengdu 610083, China; ^2^Department of Orthopedics, Chengdu Military General Hospital, Chengdu 610083, China; ^3^Department of Orthopedics, Changzheng Hospital, Second Military Medical University, Shanghai 200003, China; ^4^Department of Science and Training, Chengdu Military General Hospital, Chengdu 610083, China; ^5^Department of Training, Second Military Medical University, Shanghai 200433, China

## Abstract

Between June 2010 and June 2011, 176 patients were divided into 2 groups: a group with spinal metastasis of solid tumors (*n* = 157) and a group with multiple myeloma (*n* = 19). Both groups were further divided into 2 subgroups: a group receiving zoledronic acid before surgery and a control group. The zoledronic acid subgroup of the solid tumors group was group A (*n* = 81), the control subgroup of the solid tumors group was group B (*n* = 76), the zoledronic acid subgroup of the multiple myeloma group was group C (*n* = 10), and the control subgroup of the multiple myeloma group was group D (*n* = 9). The average intraoperative blood loss during spinal surgery was as follows: 1311 ± 691 mL in group A and 1752 ± 740 mL in group B (*P* = 0.000) and 1994 ± 810 mL in group C and 3134 ± 795 mL in group D (*P* = 0.000). Patients receiving zoledronic acid before surgery had significantly less intraoperative bleeding than those who did not receive it. Preoperative use of zoledronic acid can effectively reduce intraoperative bleeding during surgery for the treatment of spinal tumors.

## 1. Introduction

In clinical practice, spinal tumors are not commonly encountered. Because of the distinct characteristics of spinal anatomy and the unique epidemiological characteristics of the lesions, their diagnosis and treatment are different from those for other types of cancer. Treatments include surgery, radiotherapy, and chemotherapy. The purposes of surgery are to remove the lesion, to relieve spinal cord and nerve compression and the pain it causes, to maintain or restore spine stability, to prevent paralysis, and to improve bladder and bowel functions. Other treatments such as radiotherapy, chemotherapy, hormone therapy, and combined therapy cannot serve those purposes, and thus surgery is often performed in conjunction with those other treatments.

With the development of new surgical techniques and internal fixation, the number of indications for surgery for spinal tumors is expanding, as is the number of patients who can benefit from such surgery. However, decreasing the incidence of intraoperative hemorrhage is still a challenge. Although preoperative embolization and hemostatic drugs have been used successfully for some time now, it is necessary in the case of spinal metastasis and multiple myeloma to find more effective hemostatic control methods. At present, bisphosphonates are widely used in the treatment of cancer [1, 2], and zoledronic acid, a third-generation bisphosphonate, is commonly prescribed. Studies of zoledronic acid have mainly focused on the prevention and treatment of bone metastasis, pain control, and delaying the onset of skeletal complications [3–5]. To our knowledge, there have been no reports regarding studies of the drug's effectiveness in reducing bleeding during spinal tumor surgery.

We conducted a prospective cohort study in Shanghai Changzheng Hospital from June 2010 to June 2011 to measure the capability of zoledronic acid to reduce bleeding during spine surgery.

## 2. Materials and Methods

### 2.1. Patients

**Figure 1:**
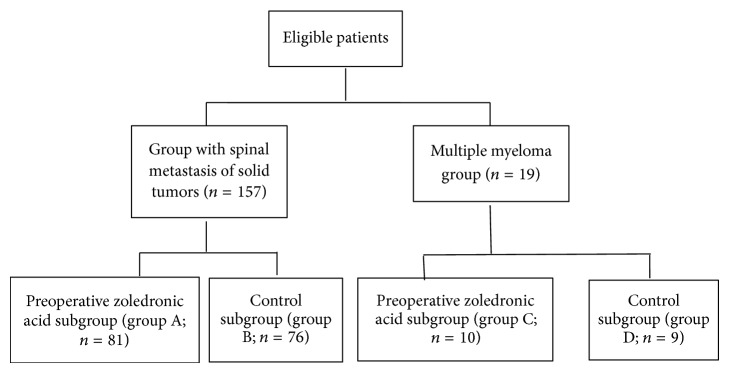
Study profile.

We chose to study patients with spinal metastases because these lesions often bleed copiously during surgery. One hundred seventy-six patients who presented between June 2010 and June 2011 at Changzheng Hospital of the Second Military Medical University in Shanghai, China, were eligible for our prospective cohort study (Figure 1). Inclusion criteria were as follows: age ≥18 years; spinal metastasis of solid tumors or multiple myeloma, with at least one neurological sign or spinal symptom; spinal cord compression restricted to a single area (but the area could include several contiguous vertebral segments); no prior treatment with bisphosphonates; and an estimated survival time of >3 months. Exclusion criteria were as follows: the presence of a primary spinal tumor; a history of previous surgery for a spinal tumor; complete paraplegia for >72 hours before study entry; a history of oral trauma; a history of radiation therapy; a history of palliative surgery (curettage and incomplete resection); health too poor for the patient to undergo surgery (especially in the presence of poor liver function, poor renal function, and cardiac dysfunction).

All eligible patients were first separated into 2 groups: a group with spinal metastasis of solid tumors (*n* = 157) and a multiple myeloma group (*n* = 19). Each of the 2 groups was then divided into a preoperative zoledronic acid subgroup and a control subgroup. The zoledronic acid subgroup of the solid tumors group was designated as group A (*n* = 81), the control subgroup of the solid tumors group was designated as group B (*n* = 76), the zoledronic acid subgroup of the multiple myeloma group was designated as group C (*n* = 10), and the control subgroup of the multiple myeloma group was designated as group D (*n* = 9). Complete information about the study protocol was provided to all patients, and all of them in turn provided written confirmation of consent to participate.

### 2.2. Procedures

#### 2.2.1. Zoledronic Acid Therapy

All study participants underwent surgical treatment and zoledronic acid therapy. The difference between groups was that in order for us to observe whether zoledronic acid reduced intraoperative bleeding we gave the drug (4 mg via 15-minute infusion) to patients in groups A and C 2 to 5 days before surgery, and we gave the same amount to patients in groups B and D after surgery.

#### 2.2.2. Surgical Treatment

Two weeks before surgery, we stopped giving patients antiplatelet drugs and other drugs, including aspirin, nonsteroidal anti-inflammatory drugs, clopidogrel, and steroids, to avoid prolonging coagulation time and bleeding time during surgery. Routine blood tests and biochemical tests were performed before drug withdrawal and before surgery. If a patient's coagulation time was beyond the normal range, surgery was delayed or canceled.

Preoperative data from computed tomography or magnetic resonance imaging were used to determine the spatial location of patients' tumors and to plan surgery. The surgical approaches that we used varied according to tumor location: posterior, anterior, and a combination. All patients underwent aggressive excision of their lesion on the basis of the Weinstein-Boriani-Biagini staging system [6]. We filled residual cavities with autologous bone or bone cement, and we used fixation devices to restore spine stability. To reduce intraoperative bleeding, all patients underwent arterial embolization no later than 48 hours before surgery. The surgical method used differed according to each patient's level of neurological function and the size and/or the location of the spinal tumor. All surgical procedures were performed by the same group of surgeons. There was no particular restriction on choice of surgical approach or internal fixation devices.

### 2.3. Statistical Analysis

Descriptive statistics (mean, standard deviation, and range) were used to characterize the eligible patients at baseline. The Student's *t*-test was used to compare continuous data such as age and amount of intraoperative bleeding between the preoperative zoledronic acid subgroups and the control subgroups. The chi-square test was used to compare categorical data such as patients' sex, preoperative Frankel score [7], location of primary tumor, number of spinal metastases, tumor location, surgical approach, and arterial embolization (yes/no) between the preoperative zoledronic acid subgroups and the control subgroups. A *P* value of 0.05 was considered statistically significant. All statistical analyses were performed using the SPSS software package (version 16.0; IBM, Armonk, NY, USA).

## 3. Results

### 3.1. Baseline Characteristics of Spinal Metastasis of Solid Tumors

There were no statistically significant differences in demographic or clinical characteristics between group A and group B at baseline (Table 1). In group A, the mean age was 54.6 ± 10.1 years, and the ratio of men to women was 16 : 11. In group B, the mean age was 52.5 ± 11.0 years, and the ratio of men to women was 23 : 15. Primary solid tumors included lung cancer, liver cancer, thyroid cancer, renal cell carcinoma, breast cancer, prostate cancer, gastric cancer, nasopharyngeal cancer, cervical cancer, esophageal cancer, and colorectal cancer. Lung and liver cancer made up the largest proportion of tumors, at 31.2% and 19.1%, respectively. Frankel scores were used to assess patients' neurological impairment. As scores move from E to A, there is increasingly impaired neurological function [7]. Before surgery, 10 patients (12.3%) in group A and 5 patients (6.6%) in group B had no perceivable neurological impairment (Frankel score E), 36 patients (44.4%) in group A and 46 patients (60.5%) in group B had some neurological impairment and could still move their lower limbs and walk (Frankel score D), 26 patients (32.1%) in group A and 20 patients (26.3%) in group B had some motor function preserved but could not take advantage of it (Frankel score C), 6 patients (7.4%) in group A and 4 patients (5.3%) in group B had preserved sensation without motor function (Frankel score B), and 3 patients (3.7%) in group A and 1 patient (1.3%) in group B had complete neurological injury—no motor or sensory function at all (Frankel score A). Preoperative arterial embolization was performed in 61 patients (75.3%) in group A and in 60 patients (78.9%) in group B. Tumors were cervical in 14 patients, thoracic in 35, and lumbar in 32 in group A and were cervical in 17, thoracic in 34, and lumbar in 25 in group B, a distribution that did not represent a statistically significant difference between groups.

### 3.2. Baseline Characteristics of Multiple Myeloma

There were no statistically significant differences in demographic or clinical characteristics between group C and group D at baseline (Table 2). In group C, the mean age was 63.8 ± 4.5 years, and the ratio of men to women was 7 : 3. In group D, the mean age was 61.7 ± 3.7 years, and the ratio of men to women was 2 : 1. All 19 patients underwent arterial embolization before surgery. In group C, 4 patients had single spinal metastases and 6 had multiple metastases; in group D, 5 patients had single spinal metastases and 4 had multiple metastases. There was no statistically significant difference between the 2 groups in terms of Frankel score (Table 2). Tumors were cervical in 2 patients, thoracic in 5, and lumbar in 3 in group C and were cervical in 3, thoracic in 4, and lumbar in 2 in group C, a distribution that did not represent a statistically significant difference between groups.

### 3.3. Intraoperative Blood Loss and Surgical Approach in Patients with Spinal Metastasis of Solid Tumors

The surgical approaches used varied according to tumor location: posterior, anterior, and a combination.  Table 3 shows the distribution of cases in 2 groups of patients with different surgical approaches. There was no statistically significant difference between groups regarding surgical approaches.

The amount of intraoperative blood loss was defined as the quantity of blood in suction canisters minus the volume of irrigation solution, plus the blood loss estimated by the difference in weights of dry versus blood-soaked surgical sponges [8]. The average intraoperative blood loss was 1311 ± 691 mL (range, 380–3400 mL) in group A and 1752 ± 740 mL (range, 450–4200 mL) in group B (*P* = 0.000). In group A, blood loss was <500 mL in 10 patients (12.3%), between 500 and 1000 mL in 26 patients (32.1%), between 1001 and 1500 mL in 17 patients (21.0%), between 1501 and 2000 mL in 18 patients (22.2%), and >2001 mL in 10 patients (12.3%). The corresponding losses in group B occurred in 3 patients (3.9%), 8 patients (10.5%), 20 patients (26.3%), 25 patients (32.9%), and 20 patients (26.3%), respectively. There were statistically significant differences in blood loss between the 2 groups (*P* = 0.001). Patients in the metastasis group who received zoledronic acid before surgery (group A) had significantly less bleeding than did patients in the control group (group B; Table 3).

### 3.4. Intraoperative Blood Loss and Surgical Approach in Patients with Multiple Myeloma

The average intraoperative blood loss was 1994 ± 810 mL (range, 890–3200 mL) in group C and 3134 ± 795 mL (range, 2200–4450 mL) in group D (*P* = 0.000). In group C, blood loss was between 500 and 1000 mL in 2 patients (20.0%), between 1001 and 2000 mL in 4 patients (40.0%), between 2001 and 3000 mL in 3 patients (30.0%), and >3001 mL in 1 patient (10.0%). The corresponding losses in group D occurred in 0 patients (0.0%), 0 patients (0.0%), 5 patients (55.6%), and 4 patients (44.4%), respectively. There were statistically significant differences in blood loss between the 2 groups (*P* = 0.041). Patients in the multiple myeloma group who received zoledronic acid before surgery (group C) had significantly less bleeding than did patients in the control group (group D; Table 4).

Five of 19 patients with multiple myeloma underwent surgery that used an anterior approach; in the other 14, the approach was posterior. There was no statistically significant difference between group C and group D regarding surgical approaches.

## 4. Discussion

Because of their location and rapid progression, spinal tumors can compress the spinal cord or cause a pathological fracture that injures the cord. When this occurs, surgical excision of the tumor is the only treatment that can relieve symptoms. Many previous studies have shown that even for patients with metastatic spinal tumors surgery can still improve quality of life [9]. Extensive surgical procedures in patients with spinal tumors are often complicated by excessive blood loss [10], but we believe that reducing the amount of intraoperative bleeding can allow better visualization, providing the spine surgeon with more time to determine the scope of tumor involvement. In addition, a reduction in bleeding allows for better protection of the surrounding normal tissue through avoidance of tumor contamination, creation of a more definite tumor resection boundary, a decrease in procedure duration, and improved safety. Through clinical practice and case preliminary review, we stumbled onto the possibility that there was common factor involved when patients had reduced intraoperative bleeding—preoperative use of zoledronic acid. To verify whether this was the case in patients undergoing surgery for spinal tumors, we designed and implemented a prospective cohort study.

We chose patients with either spinal metastasis or multiple myeloma for our study because that population frequently experiences intraoperative bleeding despite any hemostatic measures taken. Because of their abundant blood supply, metastatic tumors bleed easily during resection [11, 12]. Osteolytic lesions and a hemorrhagic tendency are prominent characteristics of multiple myeloma. The tendency to hemorrhage is caused by a reduced platelet count and the presence of coagulation disorders. In multiple myeloma, bone trabeculae are destroyed, and there is cortical thinning, with osteoporosis making the bone soft and brittle [13]. It must be emphasized here that although the preferred treatment for multiple myeloma is radiation therapy, the initial symptoms of patients in our study with multiple myeloma were spinal, for which surgery was necessary.

Intraoperative bleeding during spinal surgery is mainly related to age, vascular conditions, the pathological types of tumors involved, tumor volume, the area involved, the extent of the surgery, and previous treatments [14, 15]. To eliminate the effect of these factors in our study, we set strict inclusion and exclusion criteria for participants. We found no statistically significant differences in demographic or clinical characteristics at baseline between the 2 solid tumor groups: group A (those given zoledronic acid before surgery) and group B (the control group). We found similar baseline results for the multiple myeloma groups: group C (those given zoledronic acid before surgery) and group D (the control group). Because of the massive bleeding associated with surgical procedures involving the spine, preoperative arterial embolization is usually imperative [16]. In our study, 20 of 81 patients (24.7%) in group A and 16 patients (21.0%) in group B either could not undergo embolization or could undergo only partial embolization. This was the case because the tumor-feeding artery also supplied the artery of Adamkiewicz. The embolization results for the 2 groups had no effect on baseline comparability.

In most cases, the amount of hemorrhaging was as follows: cervical < thoracic < lumbar and single-level segments < multiple-level single vertebral segments < several contiguous vertebral segments. We used the Weinstein-Boriani-Biagini staging system in planning surgery. To decrease the influence of tumor location (invasion) and surgical approach on intraoperative bleeding, we compared several groups (A versus B and C versus D) of patients in regard to these two variables. There were no statistically significant differences in tumor location or surgical approach between group A and group B or between group C and group D.

Because of ethical concerns, we did not use random assignment to groups. To control bias, we used blinded assignment instead. Drug-dispensing staff members and surgeons were different people. The former did not know the specific surgical method chosen, and the latter did not have access to information about group assignments. Both control groups (groups B and D) also received 100 mL infusion of physiological saline over a 15-minute period before surgery. Spine surgeons were responsible only for performing operations; blood loss estimation was done by the anesthesiologist.

It has been demonstrated that zoledronic acid affects both osteoclast inhibition and myeloma cell apoptosis. It inhibits osteoclast recruitment and maturation, prevents the development of monocytes into osteoclasts, induces osteoclast apoptosis, and interrupts osteoclasts' attachment to bone [17]. Researchers have reported that zoledronic acid can be given safely over several minutes and produce similar antiresorptive effects, as assessed by bone resorption markers [18–20]. Recent studies have also shown that zoledronic acid is effective in the treatment of multiple myeloma and some solid tumors, such as lung cancer, breast cancer, and prostate cancer, having the potential for an antitumor effect [2, 21–23].

Potential adverse events associated with zoledronic acid therapy include inflammatory reactions at the injection site, acute phase reactions after intravenous use, hyperthermia, and hypocalcemia. Additionally, renal impairment and avascular osteonecrosis of the jaw are infrequent but serious complications of this treatment [17]. Zoledronic acid can be used for a long time, but it is necessary to monitor patients' renal function. Creatinine level is one of the important indexes that reflect renal function. We routinely tested patients' renal function before and after zoledronic acid therapy and surgery. All patients who did not have test values in the normal range for preoperative hepatic, renal, and cardiac function were excluded from our study. In addition, we used measures during surgery to prevent bleeding and protect renal function, including hypervolemic hemodilution and controlled hypotension. During anesthesia administration, we began to dilute patients' blood to maintain a hypervolemic status. We used controlled hypotension during tumor resection, maintaining a systolic pressure in the range of 80 to 90 mmHg, or a mean arterial pressure of 60 mmHg.

Zoledronic acid has been used as an adjuvant therapy for the prevention and treatment of metastatic malignant bone tumors. Guidelines suggest that the drug can be used for a long time, at a dosage of 4 mg every 28 days by intravenous infusion. However, there is no universal agreement on optimal starting time or therapy duration [2, 17, 24]. Our preliminary results support the hypothesis that if zoledronic acid is used before surgery, it confers the unexpected benefit of reduced intraoperative bleeding. Why did the preoperative administration of 4 mg zoledronic acid once every 2 to 5 days significantly decrease intraoperative bleeding in our study? We speculate that this happened because zoledronic acid inhibits osteoclast activity, enhancing the anti-invasion activity of bone stromal and epithelial cells, inhibiting osteolysis, reducing bone loss and preserving repaired bone, and inhibiting angiogenesis, thus inhibiting intraoperative hemorrhage. However, these are conjectures based on analysis of the known mechanisms of zoledronic acid. Further research is necessary to confirm the bleeding-inhibitory properties of zoledronic acid in spine surgery. Because of our findings, we believe that patients with spinal metastatic carcinoma and those with malignant primary spinal tumors should be given zoledronic acid as soon as possible. Preoperative use of zoledronic acid is especially important in patients with newly diagnosed tumors that must be treated surgically.

## Figures and Tables

**Table 1:** Baseline characteristics in 157 patients with metastases^a^.

Characteristic	Preoperative zoledronic acid group (group A; *n* = 81)	Control group (group B; *n* = 76)	*P*
Sex			0.871
Men	48	46	
Women	33	30	
Age (y)			0.205
Mean ± SD	54.6 ± 10.1	52.5 ± 11.0	
Range	33–75	31–87	
Primary tumor			0.293
Lung	30	19	
Liver	11	19	
Thyroid	14	8	
Breast	7	10	
Renal	6	7	
Prostate	6	8	
Other	7	5	
Preoperative Frankel score			0.296
A	3	1	
B	6	4	
C	26	20	
D	36	46	
E	10	5	
Number of spinal metastases			0.508
Single	74	67	
Multiple	7	9	
Complete arterial embolism			0.588
Yes	61	60	
No	20	16	
Tumor location			0.605
Cervical	14	17	
Thoracic	35	34	
Lumbar	32	25	

SD: standard deviation.

^a^The Student's *t*-test was used for continuous variables, and chi-square analysis was used for categorical variables.

**Table 2:** Baseline characteristics in 19 patients with multiple myeloma^a^.

Characteristic	Preoperative zoledronic acid group (group C; *n* = 10)	Control group (group D; *n* = 9)	*P*
Sex			0.876
Men	7	6	
Women	3	3	
Age (y)			0.283
Mean ± SD	63.8 ± 4.5	61.7 ± 3.7	
Range	55–70	57–67	
Preoperative Frankel score			0.322
A	0	0	
B	2	0	
C	2	4	
D	4	2	
E	2	3	
Number of spinal metastases			0.498
Single	4	5	
Multiple	6	4	
Tumor location			0.795
Cervical	2	3	
Thoracic	5	4	
Lumbar	3	2	

SD: standard deviation.

^a^The Student's *t*-test was used for continuous variables, and chi-square analysis was used for categorical variables.

**Table 3:** Comparison of intraoperative blood loss and surgical approach in 157 patients with metastases.

Intraoperative blood loss	Preoperative zoledronic acid group (group A; *n* = 81)	Control group (group B; *n* = 76)	*P*
Mean ± SD (volume per milliliter)	1311 ± 691	1752 ± 740	0.000
Range (volume per milliliter)	380–3400	450–4200	
Distribution			0.001
≤500 mL	10	3	
500–1000 mL	26	8	
1001–1500 mL	17	20	
1501–2000 mL	18	25	
≥2001 mL	10	20	
Surgical approach			0.704
Anterior	12	14	
Posterior	67	59	
Combined	2	3	

**Table 4:** Comparison of intraoperative blood loss and surgical approach in 19 patients with multiple myeloma.

Intraoperative blood loss	Preoperative zoledronic acid group (group C; *n* = 10)	Control group (group D; *n* = 9)	*P*
Mean ± SD (volume per milliliter)	1994 ± 810	3134 ± 795	0.007
Range (volume per milliliter)	890–3200	2200–4450	
Distribution			0.041
500–1000 mL	2	0	
1001–2000 mL	4	0	
2001–3000 mL	3	5	
≥3001 mL	1	4	
Surgical approach			0.510
Anterior	2	3	
Posterior	8	6	
Combined	0	0	
